# Multi-Trait, Multi-Environment Genomic Prediction of Durum Wheat With Genomic Best Linear Unbiased Predictor and Deep Learning Methods

**DOI:** 10.3389/fpls.2019.01311

**Published:** 2019-11-08

**Authors:** Osval A. Montesinos-López, Abelardo Montesinos-López, Roberto Tuberosa, Marco Maccaferri, Giuseppe Sciara, Karim Ammar, José Crossa

**Affiliations:** ^1^Facultad de Telemática, Universidad de Colima, Colima, Mexico; ^2^Departamento de Matemáticas, Centro Universitario de Ciencias Exactas e Ingenierías (CUCEI), Universidad de Guadalajara, Guadalajara, Mexico; ^3^Department of Agricultural and Food Sciences, University of Bologna, Bologna, Italy; ^4^Global Wheat Breeding Program, International Maize and Wheat Improvement Center (CIMMYT), Mexico City, Mexico

**Keywords:** durum wheat, deep learning, multi-trait, univariate trait, GBLUP, genomic selection

## Abstract

Although durum wheat (*Triticum turgidum* var. *durum* Desf.) is a minor cereal crop representing just 5–7% of the world’s total wheat crop, it is a staple food in Mediterranean countries, where it is used to produce pasta, couscous, bulgur and bread. In this paper, we cover multi-trait prediction of grain yield (GY), days to heading (DH) and plant height (PH) of 270 durum wheat lines that were evaluated in 43 environments (country–location–year combinations) across a broad range of water regimes in the Mediterranean Basin and other locations. Multi-trait prediction analyses were performed by implementing a multi-trait deep learning model (MTDL) with a feed-forward network topology and a rectified linear unit activation function with a grid search approach for the selection of hyper-parameters. The results of the multi-trait deep learning method were also compared with univariate predictions of the genomic best linear unbiased predictor (GBLUP) method and the univariate counterpart of the multi-trait deep learning method (UDL). All models were implemented with and without the genotype × environment interaction term. We found that the best predictions were observed without the genotype × environment interaction term in the UDL and MTDL methods. However, under the GBLUP method, the best predictions were observed when the genotype × environment interaction term was taken into account. We also found that in general the best predictions were observed under the GBLUP model; however, the predictions of the MTDL were very similar to those of the GBLUP model. This result provides more evidence that the GBLUP model is a powerful approach for genomic prediction, but also that the deep learning method is a practical approach for predicting univariate and multivariate traits in the context of genomic selection.

## Introduction

Nowadays, wheat is the most widespread crop around the world, for it is cultivated on approximately 219 million hectares. It is a basic staple food of mankind, since it provides 18% of the daily intake of calories and 20% of protein (Royo et al., 2017). In the Mediterranean region, wheat covers 27% of arable land, 60% of which is cultivated with durum wheat (*Triticum turgidum* var. *durum* Desf.) used for pasta, couscous, bulgur and bread production. Although durum represents just 5–7% of the world’s total wheat crop, it is a staple food of the Mediterranean diet, widely recognized for its health benefits and the prevention of cardiovascular disease. One reason that durum wheat is chosen for manufacturing premium pasta is that it is the hardest of all wheats due to its density, high protein content and gluten strength, which are essential features for producing firm pasta with consistent cooking quality (Royo et al., 2017).

Durum breeding based on genomics-assisted approaches will play an increasingly important role in delivering cultivars more resilient to climate change and with more nutritious and high-quality semolina (Tuberosa and Pozniak, 2014). Accordingly, some durum wheat breeding programs are adopting genomic selection (GS) to accelerate early identification and selection of superior genotypes (Michel et al., 2019; Steiner et al., 2019). GS was first proposed by Meuwissen et al. (2001) as a method for selecting candidate individuals with the help of a regression model that uses all the dense molecular markers simultaneously as independent variables; with this information, a model is trained using a reference population composed of genomic information and phenotypic information. The trained model is then used with a testing population to produce predictions of the individuals in the testing population that were not phenotyped but only genotyped. Applications of GS are common in many areas of animal and plant breeding since there is empirical evidence that this technology has the power to: (a) significantly reduce the time needed to develop new varieties or animals, (b) increase genetic gain in a shorter period of time, and (c) revolutionize the traditional way of developing plant and animals.

For durum wheat breeding, grain yield and semolina quality traits are important selection criteria usually applied and tested in late generations in relatively few lines due to high screening cost, which lowers selection efficiency due to the advancement of undesirable lines into large and expensive yield trials for grain yield and quality trait testing (Fiedler et al., 2017). For this reason, the potential application of genomic selection (GS) in a durum wheat breeding program using 1,184 durum wheat breeding lines was investigated by Fiedler et al. (2017), with prediction accuracies ranging from 0.27 to 0.66 for five quality traits, which pointed out the importance of GS for further enhancing breeding efficiency in durum cultivar development. On the other hand, Crossa et al. (2016) used a genomic marker × environment interaction model for (i) making genome-based predictions of untested individuals, as well as (ii) identifying genomic regions whose effects are stable across environments and other genomic regions that show environmental specificity. The same authors used a multi-parental durum wheat population that was evaluated for grain yield, grain volume weight, thousand-kernel weight and heading date in four environments in Italy. The marker × environment interaction model had better genomic-enabled prediction accuracy than the single-environment or across-environment models. The marker × environment model found that genes controlling heading date, *Ppd* and *FT* on chromosomes 2A, 2B and 7A, showed stable effects across environments as well as environment-specific effects. For grain yield, regions in chromosomes 2B and 7A had large marker effects.

Additionally, since more than one trait was measured in the durum wheat experiments of this study, we performed multi-trait analyses that outperformed univariate analyses. Empirical evidence indicates that multi-trait analyses outperform univariate analyses in terms of prediction performance when the correlation between traits is moderate or large (Jia and Jannink, 2012; Jiang et al., 2015; Montesinos-Lopez et al., 2016; Schulthess et al., 2018; Covarrubias-Pazaran et al., 2018; Montesinos-López et al., 2018b; Montesinos-López et al., 2019a). However, multi-trait analysis is more complex than univariate analysis and more prone to overfitting; for this reason, in many cases the prediction performance of the former is worse than that of the latter. The problem of overfitting is very challenging, not only in conventional multi-trait analysis, but also in conventional statistical and machine learning algorithms like deep learning models.

Deep learning is a generalization of artificial neural networks where the number of layers used is more than one, which helps to capture complex patterns in the data at the cost of increasing the computational resources required due to the fact that more neurons are used. It should be pointed out that deep learning methodology is being implemented in different areas of knowledge such as astrophysics (for classifying exoplanets), geology (for predicting earthquakes), information technology (for classifying emails), botany (for classifying species using photos), engineering (for developing self-driving cars) and meteorology (for predicting time series), among others. In biological sciences there are also many successful applications of deep learning (Alipanahi et al., 2015; Cole et al., 2017; Pan and Shen, 2017; Tavanaei et al., 2017), while in the context of GS, this methodology is applied to continuous (univariate and multivariate), binary and ordinal (univariate) and mixed traits (continuous, binary and ordinal) (Montesinos-López et al., 2018a; Montesinos-López et al., 2018b; Montesinos-López et al., 2019a; Montesinos-López et al., 2019b).

In order to study the feasibility of using GS methodology to select durum wheat genotypes early in time, we evaluated the prediction performance of three statistical learning methods, namely (i) the univariate best linear unbiased predictor (GBLUP), (ii) the multi-trait deep learning model (MTDL), and (iii) the univariate deep learning (UDL) model. Each type of model was evaluated taking into account genotype × environment interaction (I) and ignoring it (WI) in order to evaluate the impact of the interaction term on the prediction performance of out-of-sample genotypes not used for training the model. All these models were implemented using real data sets from multi-environment trials conducted mainly in the Mediterranean Basin.

## Materials and Methods

### Experimental Data

#### Phenotypic Data

This data set originated in durum wheat experiments conducted with (i) 189 elite cultivars collectively named the Durum Wheat Panel assembled at the University of Bologna and first described by Maccaferri et al. (2006), and (ii) an additional set of 81 elite cultivars contributed by collaborators worldwide. Field experiments were conducted mainly in Mediterranean countries, Hungary and Mexico under both rainfed and irrigated conditions. **Table 1** reports the acronyms and further details of the environments under study.

**Table 1:** Acronyms of the 43 environments and the notations used to represent them. Field trials were conducted in nine countries under rainfed (r) and/or irrigated (i) conditions in 11 years (2003-04-05-06-07-08-09-12-13-14-15).

Environment*	Notation	Environment	Notation	Environment	Notation
Hng-i12	E1	Itl5-r15	E16	Mxc-r14	E31
Hng-i13	E2	Lbn-i04	E17	Spn1-r04	E32
Hng-r12	E3	Lbn-i05	E18	Spn2-r05	E33
Hng-r13	E4	Lbn-r04	E19	Syr-i05	E34
Itl-r06	E5	Lbn-r05	E20	Syr-i06	E35
Itl1-r03	E6	Mrc-i04	E21	Syr-i07	E36
Itl1-r04	E7	Mrc-r04	E22	Syr-r05	E37
Itl1-r12	E8	Mxc-i14	E23	Syr-r07	E38
Itl1-r13	E9	Mxc-i06	E24	Syr2-r06	E39
Itl2-r04	E10	Mxc-i07	E25	Tns-i05	E40
Itl2-r05	E11	Mxc-i14	E26	Tns-r05	E41
Itl3-r08	E12	Mxc-i06	E27	Trk-i12	E42
Itl4-r07	E13	Mxc-n07	E28	Trk-r12	E43
Itl5-n15	E14	Mxc-r06	E29	–	–
Itl5-r09	E15	Mxc-r07	E30	–	–

*Hung, Hungary; Itl, Italy; Lbn, Lebanon; Mrc, Morocco; Mxc, Mexico; Spn, Spain; Syr, Syria; Tns, Tunisia; Trk, Turkey.

The analyzed traits were grain yield (GY), days to heading (DH) and plant height (PH) evaluated in 270 lines. Field trials were conducted over 11 years, and in each year only some locations were evaluated. A total of 43 environments (country-site-year combinations) were included in this study. The years under the study were 2003–2009 and 2012–2015. Other traits of interest were also measured in addition to GY, DH and PH, but due to the scarcity of the data, this study only considered these three traits and information from 43 environments. It is important to mention that the number of lines in each environment ranged from a minimum of 57 lines to a maximum of 193 lines, with a mean and median of 180.9 and 186 lines, respectively.

#### Genotypic Data

The genotypes of all 270 lines were obtained using 24,576 single nucleotide polymorphisms (SNPs) from the consensus map of tetraploid wheat assembled by Maccaferri et al. (2015) as a bridge to integrate durum and bread wheat genomics and breeding mapped in tetraploid wheat. The tetraploid consensus map incorporates SSR, DArT^®^ and SNP markers from 13 mapping populations. The SSR and DArT^®^ profiles (Mantovani et al., 2008; Maccaferri et al., 2011) were integrated with the high-density Infinium^®^ iSelect^®^ Illumina 90K SNP array (Wang et al., 2014; Maccaferri et al., 2015). DNA was extracted from a bulk of 25 one-week-old seedlings per accession using the DNeasy 96 Plant Kit (Qiagen GmbH, Hilden, Germany). The final array included 81,587 transcript-associated SNPs, 8,000 of which are durum-specific SNPs (Wang et al., 2014). Those SNPs with >10% missing values or <0.05 minor allele frequency were excluded. After line-specific quality control, 14,163 SNPs were retained.

### Statistical Models

#### Multiple-Environment Genomic Best Linear Unbiased Predictor (GBLUP) Method

The univariate GBLUP model including the random interaction term between the genomic effect of the *j*th line and the *i*th environment method is represented by the model

(1) $$y_{ij}=E_{i}+g_{j}+gE_{ij}+e_{ij}$$

where (i) *y_ij_* represents the response of the *j*th line in the *i*th environment (*i*=1,2,…,*I*, *j*=1,2,…*J*); (ii) *E_i_* denotes the fixed effect of the *i*th environment; (iii) *g_j_* represents the random genomic effect of the *j*th line, with ***g*** = $g={\left(g_{1},\ldots ,g_{J}\right)}^{T}\sim N\left(0,\sigma_{1}^{2}\;G_{g}\right)$, $\sigma_{1}^{2}$ is a genomic variance and ***G*** *_g_* is of order *J* × *J* and represents the genomic relationship matrix (GRM); ***G*** *_g_* is calculated (Van Raden, 2008) as $G_{g}=\frac{WW^{T}}{p}$, where *p* denotes the number of markers and ***W*** is the matrix of markers of order *J* × *p*; the ***G*** *_g_* matrix is constructed using the observed similarity at the genomic level between lines, rather than the expected similarity based on pedigree (iv) g*E_ij_* is the random interaction term between the genomic effect of the *j*th line and the *i*th environment with $gE={\left(gE_{11},\ldots ,gE_{IJ}\right)}^{T}\sim N\left(0,\sigma_{2}^{2}\;{\text{I}}_{I}⊗G\right)$, where $\sigma_{2}^{2}$ is an interaction variance; and (v) *e_ij_* is a random residual associated with the *j*th line in the *i*th environment distributed as $N\left(0,\;\sigma^{2}\right),$ where $\sigma^{2}$ is the residual variance.

#### Multi-Trait Deep Learning Method

**Figure 1:**
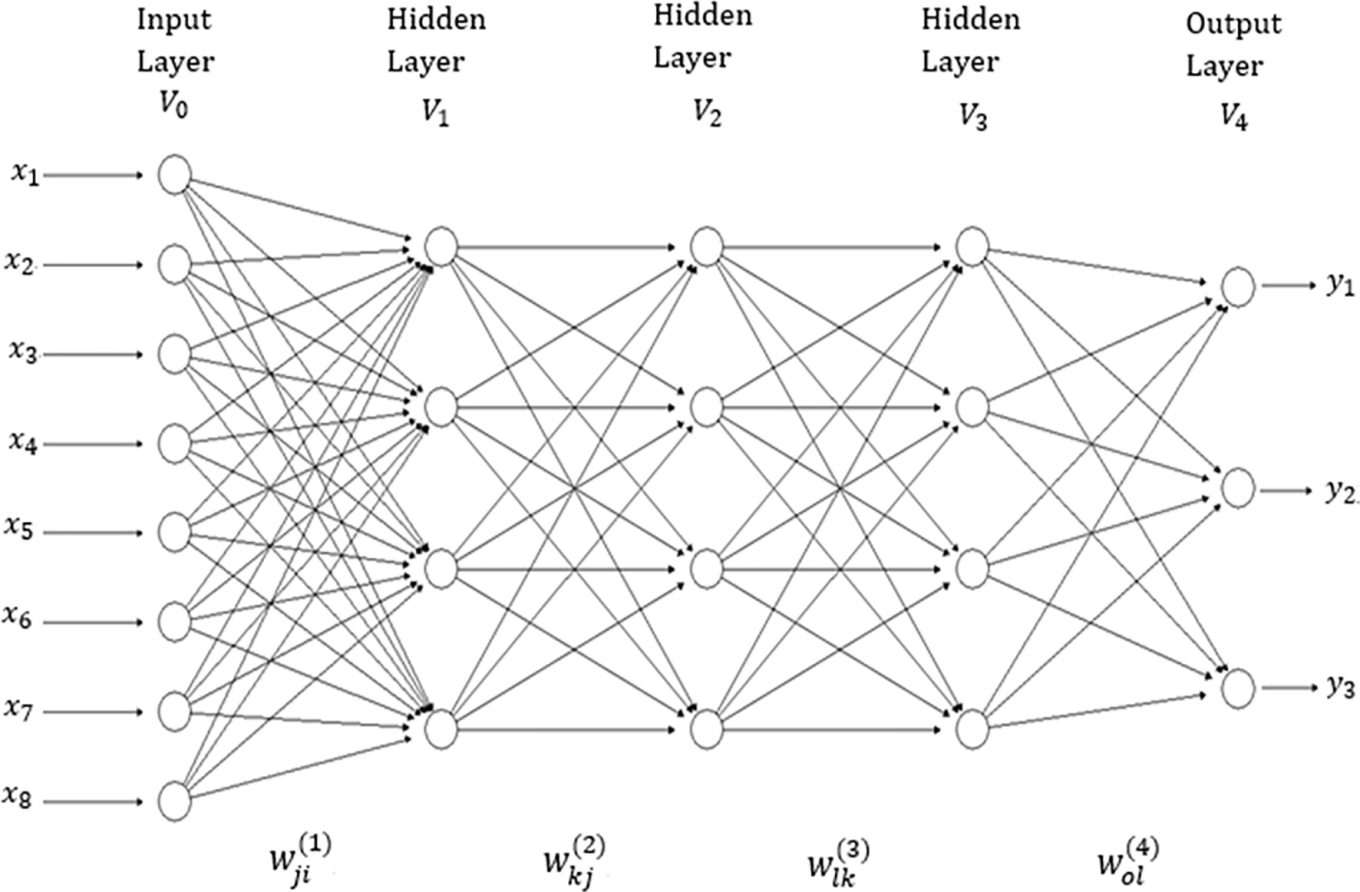
A feedforward deep neural network with one input layer, three hidden layers and one output layer. There are eight neurons in the input layer that corresponds to the input information, four neurons in each of three hidden layers, while there are three neurons in the output layer that correspond to the number of traits to be predicted.

There are many network topologies in deep learning; however, in this application we used densely connected networks, also known as feed-forward networks (see **Figure 1**). This network topology is a typical feedforward neural network (where there are no feedback interconnections) and a specific structure is not assumed in the input features (Goodfellow et al., 2016). This network consists of an input layer, an output layer and multiple hidden layers between the input and output layers. The number of features correspond to the input layer neurons (units). Hidden layer neurons perform non-linear transformation on the original input attributes (Lewis, 2016). The number of output neurons depends on the number of response variables to be predicted for continuous response variables (traits in plant breeding) which receive as input the output of hidden neurons, and produce as output the prediction values of interest (Goodfellow et al., 2016). The connected neurons form the network and the strength of the weights of each connection controls the contribution of each neuron. We implemented a type of regularization called “dropout”, which consists of temporarily removing (setting to zero) a random subset (%) of neurons and their connections during training.

The network in **Figure 1** has four layers (*V*_0_,*V*_1_,*V*_2_,*V*_3_ and *V*_4_); *V*_0_ represents the input layer (which is not counted), *V*_1_ to *V*_3_ are the hidden layers and *V*_4_ denotes the output layer. This means that the “depth” of the network is four. The size of the network given in **Figure 1** is $\left|V\right|=\left|\cup_{t=0}^{T}\right|=\left|9+5+5+5+3\right|=27$. In each layer, a +1 was added to the observed unit to represent the lacking node of the bias (or intercept). The width of the network given in **Figure 1** is *max_t_*|*V_t_*|=9. It is important to point out that the networks implemented in this research are similar to the network in **Figure 1**, but with many more input and hidden neurons. In addition to implementing networks with three hidden layers, we also implemented networks with one and two hidden layers.

The analytical forms of the model given in **Figure 1** for *o* output, with *d* inputs, *M*_1_ hidden neurons (units) in hidden layer 1, *M*_2_ hidden units in hidden layer 2, *M*_3_ hidden units in hidden layer 3, and *O* output neurons, are given by the following Equations (1)–(4):

(1) $$V_{1j}=g_{1}\left(\overset{d}{\underset{i=1}{\sum }}w_{ji}^{\left(1\right)}x_{i}\right)\text{for }j=1,\ldots ,\;M_{1}$$

(2) $$V_{2k}=g_{2}\left(\overset{M_{1}}{\underset{j=1}{\sum }}w_{kj}^{\left(2\right)}V_{1j}\right)\text{for }k=1,\ldots ,\;M_{2}$$

(3) $$V_{3l}=g_{3}\left(\overset{M_{2}}{\underset{k=1}{\sum }}w_{lk}^{\left(3\right)}V_{2k}\right)\text{for }l=1,\ldots ,\;M_{3}$$

(4) $$y_{o}=g_{4}\left(\overset{M_{3}}{\underset{l=1}{\sum }}w_{ol}^{\left(4\right)}V_{3l}\right)\text{for }o=1,\ldots ,\;O$$

where Equation (1) produces the output of each of the neurons in the first hidden layer, Equation (2) produces the output of each of the neurons in the second hidden layer, Equation (3) produces the output of each of the neurons in the third hidden layer and, finally, Equation (4) produces the output of each response variable of interest. The learning process is carried out with the weights ($w_{ji}^{\left(1\right)},w_{kj}^{\left(2\right)},w_{lk}^{\left(3\right)}\;$] and $w_{ol}^{\left(4\right)})$) that correspond to the input, to the first hidden layer, second hidden layer, third hidden layer and the output hidden layer, respectively. All the activation functions used (*g*_1_, *g*_2_, *g*_3_ and *g*_4_) in this paper were the rectified linear activation unit (RELU) function, since the response variables we wish to predict are all continuous. In Equations (1) to (4), the intercepts or bias terms were ignored.

The input variables of the multi-trait deep learning model (MTDL) were the result of concatenating the information of (i) environments, (ii) markers through the Cholesky decomposition of the genomic relationship matrix, and (iii) genotype × environment interaction (*G*×*E*). This meant that first we built the design matrices of environments (***Z*** *_E_*), genotypes (***Z*** *_G_*) and *G*×*E* (***Z*** *_GE_*), followed by the Cholesky decomposition of the genomic relationship matrix (***G***). After that, the design matrix of genotypes was post-multiplied by the transpose of the upper triangular factor of the Cholesky decomposition (***Q*** *^T^*), $Z_{G}^{*}=Z_{G}Q^{T}$, followed by the calculation of the *G*×*E* term as the product of the design matrix of the *G*×*E* term post-multiplied by the Kronecker product of the identity matrix of order equal to the number of environments and ***Q*** *^T^*, that is, $\;Z_{GE}^{*}=Z_{GE}(I_{I}⊗Q^{T})$. Finally, the matrix with input covariates used for implementing both deep learning models was equal to $X=\left[Z_{E},\;Z_{G}^{*},\;Z_{GE}^{*}\right]$.

As discussed by Montesinos-López et al. (2018a), appropriate selection of hyper-parameters is fundamental for successfully implementing deep learning models. For this reason, we used the grid search method for tuning the required number of neurons and epochs. That is, we discretized these hyper-parameters into a desired set of values of interest, and the models were trained and evaluated for all combinations of these values (that is, a “grid”); from there we selected the best combination of each hyper-parameter. The values of units used for the grid search were 20 to 200 with increments of 20, for the number of epochs we used 200 with increments of 1, while for layers we used 1, 2 and 3. This meant that the grid search consisted of 6,000 combinations of units, epochs and layers. For the remaining hyper-parameters, we chose their values according to a literature review. The percentage of dropouts used in our application was 30% (Srivastava et al., 2014; Chollet and Allaire, 2017).

#### Univariate Deep Learning

It is important to point out that a univariate deep learning (UDL) version of the multi-trait deep learning model described above was implemented, but with a feedforward neural network with only one neuron in the output layer, which meant that three independent UDL models were implemented for the three traits under study. For comparison purposes, the three models (GBLUP, MTDL and UDL) were implemented with (I) and without (WI), the interaction term. The predictor was composed of the main effects due to environments, lines and the genotype × environment (G×E) interaction term, while in the last scenario the G×E was ignored.

### Evaluating Prediction Performance With Cross-Validation

For evaluating the prediction accuracy of the Bologna data set under the three models (GBLUP, MTDL and UDL), we implemented cross-validation. The implemented random cross-validation is denoted as CV1 and consists of dividing the whole data set into a training (TRN) and a testing (TST) set. The percentages of the whole data set assigned to the TRN and TST sets were 80 and 20%, respectively. In this cross-validation, some individuals can never be part of the training set. Our random CV1 used sampling with replacement, which means that one observation can appear in more than one partition. The design we implemented mimics a prediction problem faced by breeders in incomplete field trials where lines are evaluated in some, but not all, target environments. More explicitly, TRN–TST partitions were obtained as follows: since the total number of records per trait available for the data set with multi-environments is *N*=*J*×*I*, to select lines in the TST data set, we fixed the percentage of data to be used for TST (PTesting = 20%). Then we chose 0.20×N (lines) at random, and subsequently we randomly picked one environment per line from *I* environments. The resulting cells (*ij*) were assigned to the TST data set, while cells not selected through this algorithm were allocated to the TRN data set. Lines were sampled without replacement if *J* ≥ 0.20×N, and with replacement otherwise (López-Cruz et al., 2015). For each data set under CV1, five random partitions were implemented, and with the observed and predicted values of each testing data set, we calculated the mean arctangent absolute percentage error (MAAPE) as a measure of prediction accuracy (Kim and Kim, 2016) (the smallest MAAPE indicates the best genome-based prediction model). The MAAPE is defined as the arctan of the absolute value of the difference between the observed value minus the predicted value divided by the observed value. Its main advantage is that it is defined in radians and therefore scale-free with the acceptance of missing values, and that it approaches Pi over 2 when dividing by zero.

All the analyses done were implemented in the R statistical software (R Core Team, 2019) using the keras library for implementing the DL method (Chollet and Allaire, 2017) and the BGLR library for implementing the GBLUP model (de los Campos and Pérez-Rodríguez, 2014).

## Results

**Figure 2:**
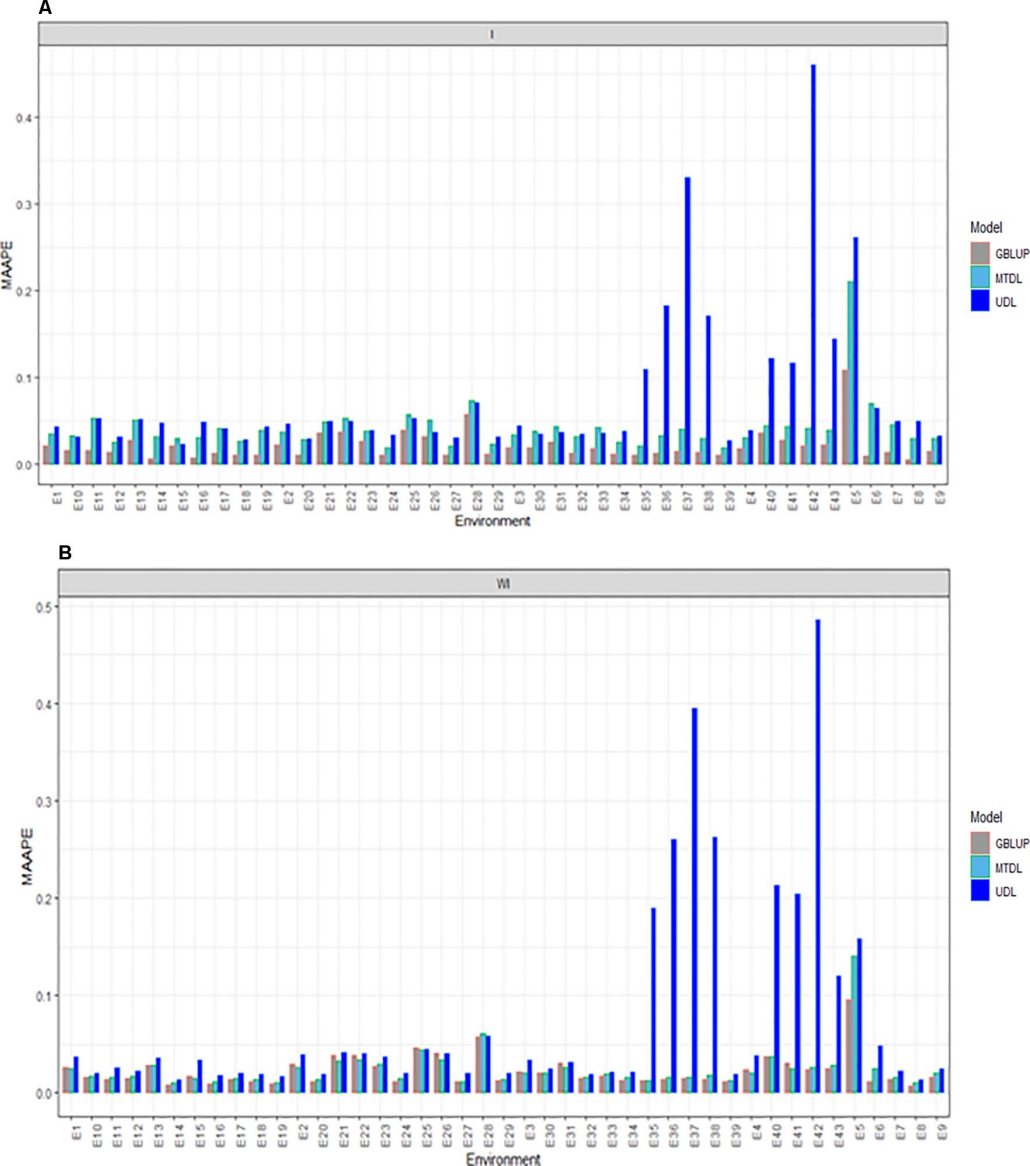
Prediction accuracy of GBLUP, MTDL and UDL in terms of MAAPE for DH in 43 environments (E1–E43) **(A)** including genotype × environment interaction (I), and **(B)** without genotype × environment (WI).

**Figure 3:**
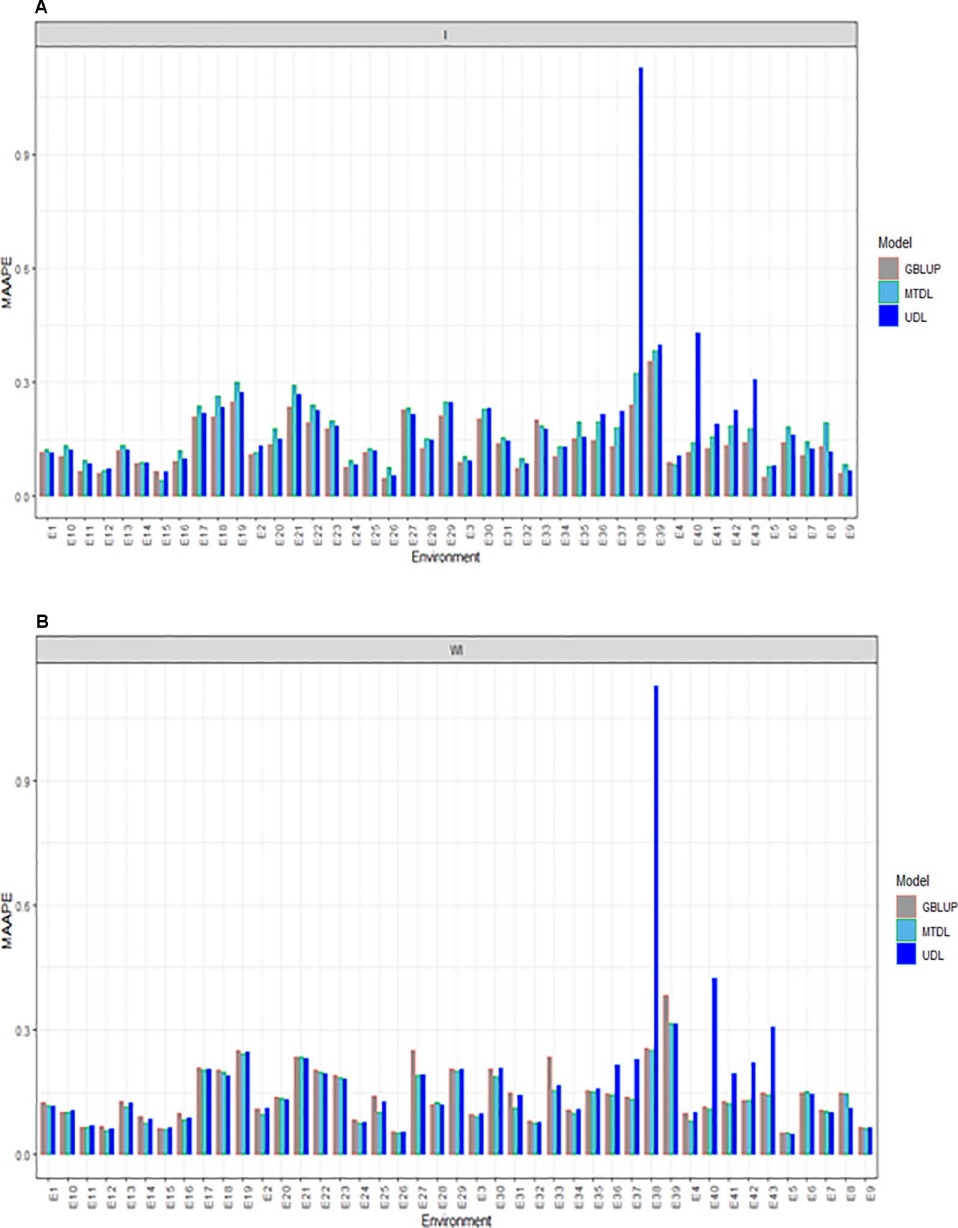
Prediction accuracy of GBLUP, MTDL and UDL in terms of MAAPE for GY in 43 environments (E1–E43) **(A)** including genotype × environment interaction (I), and **(B)** without genotype × environment (WI).

**Figure 4:**
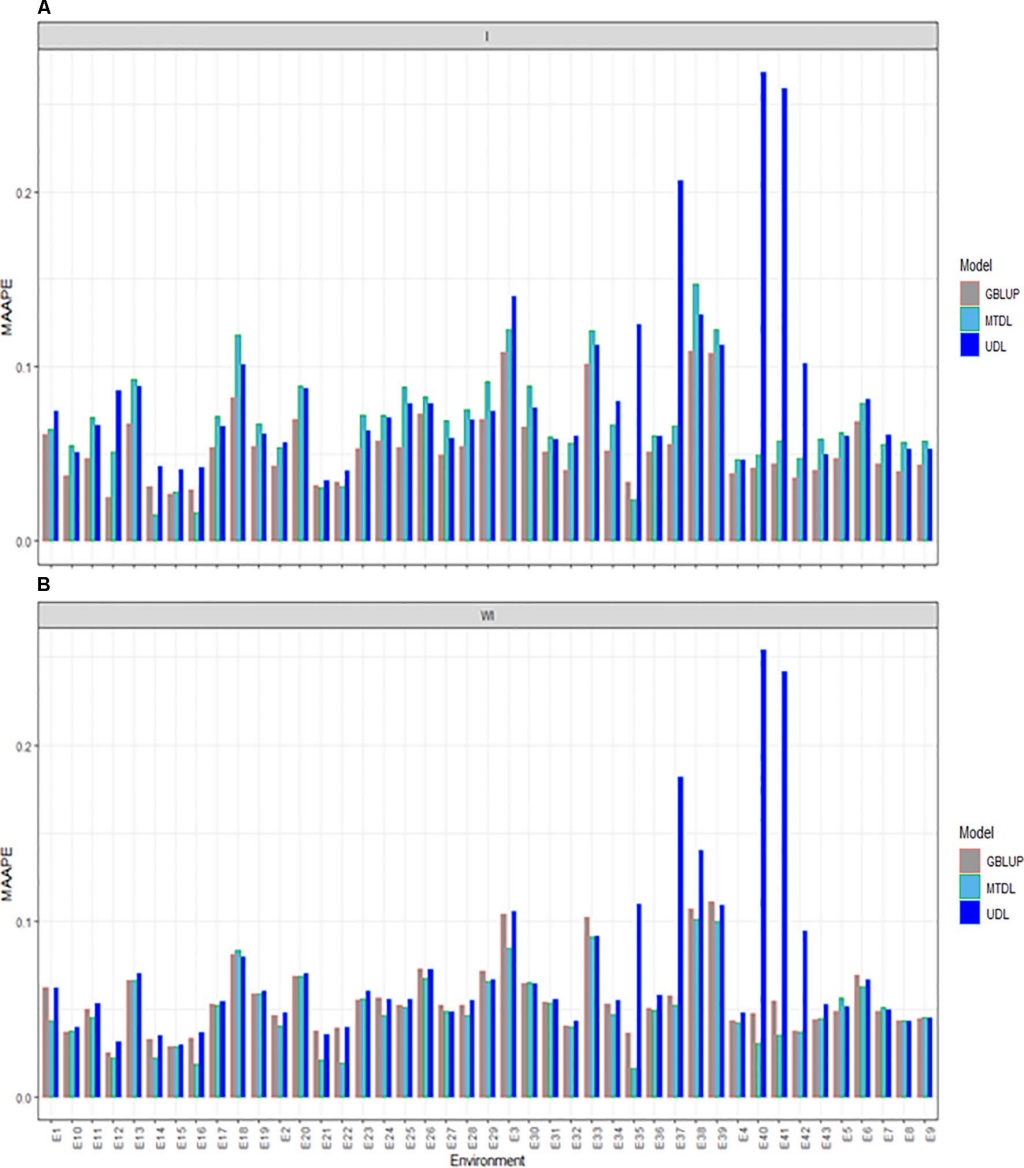
Prediction accuracy of GBLUP, MTDL and UDL in terms of MAAPE for PH in 43 environments (E1–E43) **(A)** including genotype × environment interaction (I), and **(B)** without genotype × environment (WI).

**Figure 5:**
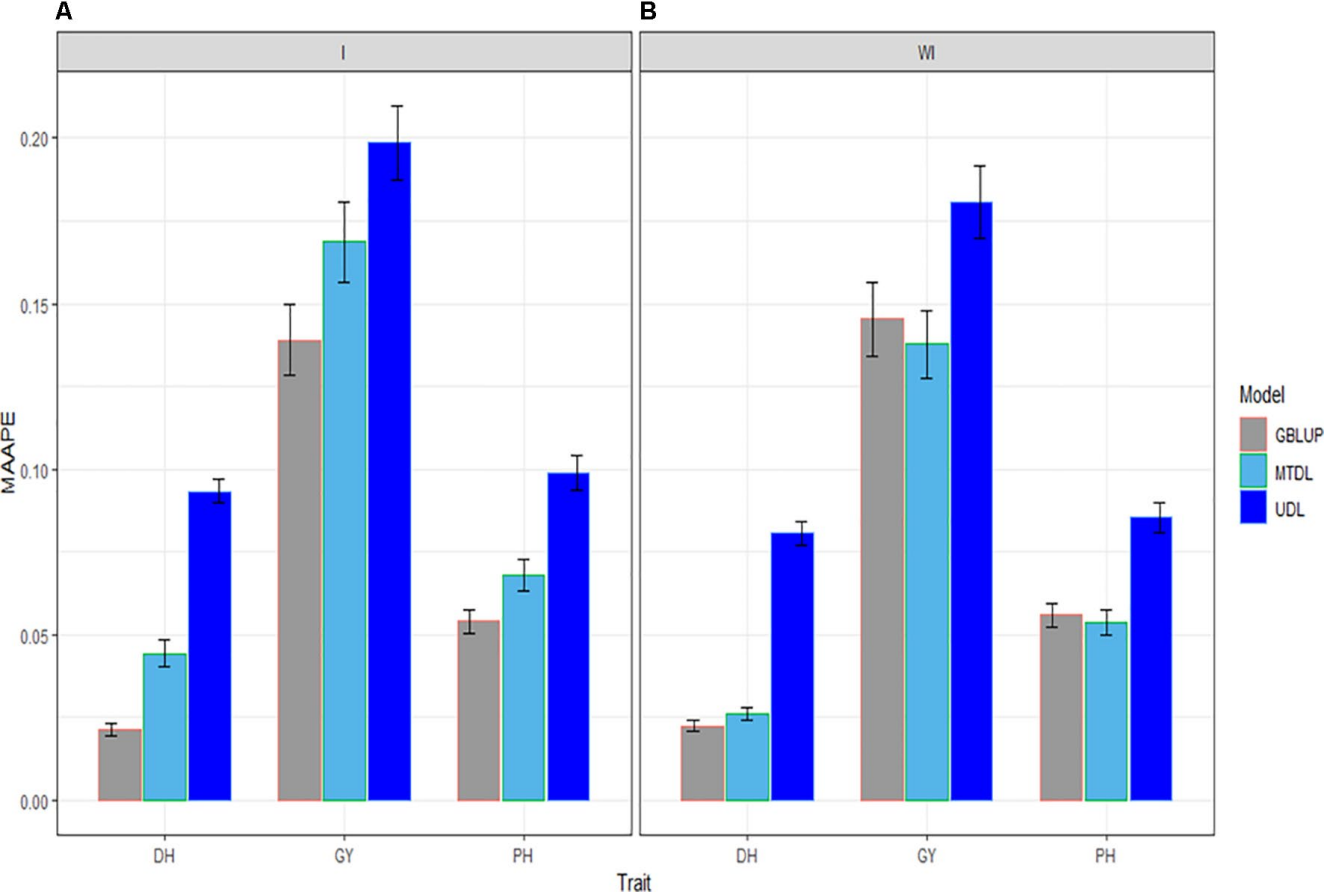
Prediction accuracy of GBLUP, MTDL and UDL in terms of average MAAPE for traits DH, GY and PH across 43 environments (E1–E43) **(A)** including genotype × environment interaction (I), and **(B)** without genotype × environment (WI).

The results are given in four sections: (1) prediction performance for DH, (2) prediction performance for GY, (3) prediction performance for PH and (4) prediction performance across environments for the three traits. Data on the genome-based predictive values using the MAAPE criterion are displayed in **Figures 2**–**5**. The same results used to construct these figures are included in the Supplemental Material at the following link: http://hdl.handle.net/11529/10548262.

### Prediction Performance for DH

Here we report the prediction accuracy in terms of MAAPE obtained by implementing the random cross-validation with 80% TRN and 20% TST for each of the three methods, GBLUP, MTDL and UDL. Each method was implemented with (I) and without (WI) interaction. With interaction (I) under MAAPE, the predictions of the three methods ranged between 0.00550 and 0.4602, with a mean and median equal to 0.0469 and 0.0334, respectively. **Figure 2A** shows that in all cases the GBLUP model was better than the other two deep learning models; the MTDL model was the second best model, while the worst predictions were those of the UDL model.

Without (WI) the interaction term, we found that the range of MAAPE was between 0.0066 and 0.4856, with a mean and median equal to 0.0409 and 0.0211, respectively. **Figure 2B** indicates that the GBLUP model was also better than the deep learning methods (MTDL and UDL), but in 14 out of 43 environments, the MTDL outperformed the GBLUP model. It is important to point out that with and without genotype ×environment interaction, the worst predictions of the three methods were found under the UDL model in environments E35–38, E5–E6 and E40–E43.

### Prediction Performance for GY

For GY, we provide the prediction performance under three methods (GBLUP, MTDL and UDL) taking into account the interaction term (I) and ignoring it (WI). Under I (interaction term) we found that the predictions in terms of MAAPE ranged between 0.0418 and 1.127, with a mean and median of 0.1652 and 0.1397, respectively. Also for GY, the best predictions were observed under the GBLUP model and the worst under the UDL model, while the predictions of the MTDL model were quite similar to those of the GBLUP model. However, in all environments, the best predictions were observed under the GBLUP model (**Figure 3A**). When the interaction term was ignored (WI), GY predictions under the MAAPE metric ranged between 0.0512 and 1.1269, with a mean and median equal to 0.1522 and 0.1287, respectively (**Figure 3B**). However, now the best predictions were observed under the MTDL model, since in 40 out of 43 environments, it outperformed the GBLUP model, and in 32 out of 43 environments, it outperformed the UDL model. It is important to point out that the worst predictions of all methods with and without the interaction term were found under the UDL model in environments E38 and E40–E43.

### Prediction Performance for PH

The three methods under study (GBLUP, MTDL and UDL) were compared for PH with (I) and without (WI) the interaction term. With the interaction term, we found that the predictions under the MAAPE metric ranged between 0.0148 and 0.2685, with a mean and median equal to 0.0687 and 0.0604, respectively. The best predictions were observed under the GBLUP model, since it was superior to the MTDL model in 38 out of the 43 environments, and in all 43 environments was better than the UDL model (**Figure 4A**). When the interaction term was ignored (WI), we found that the MAAPE metric ranged between 0.0165 and 0.2541, with the mean and median equal to 0.0598 and 0.0526, respectively (**Figure 4B**). However, now the best predictions were observed under the MTDL model in 33 out of 43 environments compared to the GBLUP model, and in 38 out 43 environments compared to the UDL model. Finally, it is important to point out that in general the worst predictions were under the UDL model, since under this method the worst predictions without genotype by environment interaction were observed in environments E35, E37–E38 and E40–42, while with the interaction term, these environments were the worst, except environment E38.

### Prediction Performance Across Environments for the Three Traits

In this subsection we provide a summary of the prediction performance of the three traits across environments using GBLUP, MTDL and UDL. When the interaction term (I) was taken into account, in **Figure 5** it is clear that the best predictions were observed under the GBLUP model and the worst under the UDL model, and the second best under the MTDL method. However, when the interaction term was ignored, the best predictions were observed under the GBLUP method and MTDL model and the worst under the UDL model; non-relevant differences can be observed in the predictions between the GBLUP and MTDL. Finally, the best predictions for DH were slightly better under the GBLUP method with the interaction term compared to the MTDL method (without the interaction term), while for traits GY and PH, there were no differences between the predictions of the GBLUP (with the interaction term) and the MTDL (without the interaction term) models.

## Discussion

Genomic selection is not new in the context of durum wheat breeding, and interest in applying this metodology for developing new varieties of durum wheat continues (Michel et al., 2019; Steiner et al., 2019). For this reason, in this study we applied GS to durum wheat with the purpose of studying the usefulness of this methodology for choosing candidate genotypes for early selection. The prediction performance of GS depends on many factors such as: (1) the genetic architecture of the target traits, (2) the number of traits under study, (3) the statistical or machine learning models used, (4) the quality of the marker information, (5) the strength of the relationship between individuals and (6) the relationship between the reference population and the validation population, among others. For this reason, in this study we evaluated the prediction performance of 270 durum wheat lines in 43 environments for traits DH, GY and PH.

Among the GWAS of durum wheat, only a few have investigated genomic predictions for grain yield and quality traits (Asbati et al., 2000; Patil et al., 2012; Crossa et al., 2017; Fiedler et al., 2017; Sukumaran et al., 2018a; Sukumaran et al., 2018b; Johnson et al., 2019; Merida-Garcıa et al., 2019). Maccaferri et al. (2011) used SSR markers and the data of a panel of 189 elite cultivars evaluated in 15 field trials in different Mediterranean countries to dissect the genetic basis of durum wheat plasticity across a broad range of water availability during the crop cycle (from 146 to 711 mm). More recently, the GWAS carried out by Sukumaran et al. (2018a; 2018b) used DArTseq markers to evaluate CIMMYT durum wheats grown under three different conditions (yield potential, drought and heat stresses). Among these studies, those of Fiedler et al. (2017), Crossa et al. (2017) and Sukumaran et al. (2018b) have shown promising genome-enabled prediction accuracy for grain yield and other traits. Notably, Crossa et al. (2017) concluded that genomic selection models incorporating marker × environment interaction are useful in durum wheat breeding for increasing genetic gains in rapid cycle selection. The present study further supports the effectiveness of GS in durum wheat.

The results obtained with GBLUP, MTDL and UDL show that the best predictions were found under the GBLUP model, followed by the MTDL model, while the worst predictions were observed under the UDL model. These results are in agreement with those reported by Montesinos-López et al. (2018a), Montesinos-López et al. (2018b) and Montesinos-López et al. (2019a), Montesinos-López et al. (2019b) who compared GBLUP models against deep learning models. Our results are also in agreement with those of Bellot et al. (2018), who found that conventional genomic prediction models outperformed deep learning methods.

Collectively, our results support the feasibility of applying genomic selection as a cost-effective means to enhance genetic gain for complex and expensive-to-measure traits in durum wheat, in agreement with Fiedler et al. (2017) and Haile et al. (2018). However, unlike the results of Haile et al. (2018), the best predictions in our study were found under a univariate model (GBLUP), while they found that multi-trait models were more accurate than the univariate trait models only for grain yield. Although the low number of lines in this study might limit the performance of deep learning models (UDL and MTDL), results show that the performance of DL models is competitive and can be applied for GS in durum wheat to develop faster and more efficient genetic gains in breeding programs.

The low prediction accuracies of deep learning methods can be attributed to the fact that deep learning methods outperform other techniques in the context of large or very large data sets, but with smaller data sets, traditional statistical and machine learning algorithms are preferable. However, although the durum wheat data set used here is not large, the prediction performance of the MTDL models was very competitive compared to the GBLUP model, and most of the time outperformed the UDL model. This result can be attributed to the fact that the training process under the MTDL model is more efficient because it simultaneously uses the information of the three traits under study, which is equivalent to increasing the sample size (Montesinos-López et al., 2018b; Montesinos-López et al., 2019b). There is some evidence that deep learning methods are better than conventional statistical and machine learning methods when tackling very complex problems such as natural language processing, image classification and speech recognition. However, there is also enough evidence that DL methods are worse in linearly separable problems and in the context of small data sets.

Although the universal approximation theorem states that “a feedforward network with a single hidden layer containing a finite number of neurons can approximate any continuous function to any degree of precision” (Cybenko, 1989), in many applications it has been shown that artificial neural networks and deep learning models (with more than one hidden layer) are not very efficient in terms of prediction performance due to the fact that the required number of hidden neurons is so incredibly large that it is not possible to implement them; for this reason, these models fail to learn and generalize correctly.

In this study, we evaluated the prediction performance of durum wheat and the results obtained show that even when the number of lines under study is low, it is possible to implement the GS methodology for selecting candidate genotypes early in time. However, the results obtained depend on the type of statistical model used, since we observed that the best predictions were under the GBLUP model, followed by the MTDL model, while the worst predictions were under the UDL model. Notably, the predictions of the MTDL model were very similar to those of the GBLUP model. From all the above, it is clear that better prediction statistical machine learning models are still required for improving the efficacy of GS methodology; for this reason, other exercises for benchmarking the existing models with real data are needed and of course new statistical or machine learning models are welcome.

## Conclusions

In this study, univariate and multivariate deep learning methods were applied to evaluate the prediction performance of durum wheat. These predictions were also compared to the predictions of the GBLUP method. In general, the best predictions were observed under the GBLUP model, although the predictions of the multi-trait deep learning model were very close to those of the GBLUP model, while the univariate deep learning model provided the worst predictions. These results can be attributed in part to the fact that the durum wheat data set used is small, which did not help the performance of deep learning methods, an approach better suited to huge amounts of training data. This notwithstanding, the multi-trait deep learning model produced predictions that were close to those of the GBLUP model. However, due to the number of hyper-parameters that need to be tuned in deep learning models, their implementation remains very challenging, because the DL process is still a combination of art and science. Even with these restrictions, we are confident that deep learning has a lot of potential to be successfully applied in the context of genomic selection.

## Data Availability Statement

The datasets generated for this study are available on request to the corresponding author.

## Author Contributions

OAML and AML performed the statistical analyses of the data and wrote the first version of the article; RT, MM, GS and KA produced the data analyzed in this study; JC proposed the initial idea and provided and participated in writing the manuscript.

## Funding

We are thankful for the financial support provided by the Bill & Melinda Gates Foundation (for maize and wheat breeding programs), the CIMMYT CGIAR CRP (maize and wheat), as well the USAID projects. We acknowledge the financial support provided by the Foundation for Research Levy on Agricultural Products (FFL) and the Agricultural Agreement Research Fund (JA) in Norway through NFR grant 267806. We are equally thankful to the European project FP7-244374 (DROPS) and the Ager Agroalimentare e Ricerca—Project ‘From Seed to Pasta: Multidisciplinary approaches for a more sustainable and high quality durum wheat production’ for the financial support to implement the field trials used in this study.

## Conflict of Interest

The authors declare that the research was conducted in the absence of any commercial or financial relationships that could be construed as a potential conflict of interest.
